# Lanadelumab for prevention of attacks of non-histaminergic normal C1 inhibitor angioedema: results from the randomized, double-blind CASPIAN Study and CASPIAN open-label extension

**DOI:** 10.3389/fimmu.2025.1502325

**Published:** 2025-05-21

**Authors:** Marc A. Riedl, Petra Staubach, Henriette Farkas, Andrea Zanichelli, Hong Ren, Christina Nurse, Irmgard Andresen, Salomé Juethner, Ming Yu, Jingmei Zhang

**Affiliations:** ^1^ Division of Allergy and Immunology, Department of Medicine, University of California, San Diego, La Jolla, CA, United States; ^2^ Department of Dermatology, University Medical Center Mainz, Mainz, Germany; ^3^ Hungarian Angioedema Center of Reference and Excellence, Department of Internal Medicine and Haematology, Semmelweis University, Budapest, Hungary; ^4^ Operative Unit of Medicine, Angioedema Center, IRCCS Policlinico San Donato, San Donato Milanese, Milan, Italy; ^5^ Dipartimento di Scienze Biomediche per la Salute, University of Milan, Milan, Italy; ^6^ Takeda Development Center Americas, Inc., Lexington, MA, United States; ^7^ Takeda Pharmaceuticals International AG, Zurich, Switzerland; ^8^ Takeda Pharmaceuticals U.S.A., Inc., Lexington, MA, United States

**Keywords:** angioedema, efficacy, non-histaminergic, nC1INH, normal C1 inhibitor, lanadelumab, prophylaxis, safety

## Abstract

**Background:**

Randomized controlled trial data for non-histaminergic normal C1 inhibitor (nC1INH) angioedema prevention are lacking.

**Methods:**

Patients aged ≥12 years with investigator-confirmed non-histaminergic nC1INH angioedema were enrolled in phase III, multicenter, randomized, placebo-controlled, double-blind CASPIAN Study (NCT04206605). Patients with ≥1 investigator-confirmed angioedema attack/4 weeks during observation period were randomized 2:1 to lanadelumab 300 mg every 2 weeks or placebo. Primary efficacy outcome was investigator-confirmed angioedema attack number during the 26-week treatment period. Safety was analyzed as treatment-emergent adverse events (TEAEs). After completing the treatment period, patients could roll over to CASPIAN open-label extension (CASPIAN OLE; NCT04444895) for an additional 26-week lanadelumab treatment to assess long-term safety and efficacy.

**Results:**

A total of 77 patients (mean ± SD age of 42.8 ± 12.9 years, 80.5% women, 88.3% White) were enrolled (lanadelumab, 50; placebo, 27). Primary efficacy outcome was not different with lanadelumab versus placebo (1.82 vs. 1.78 attacks/month; rate ratio, 1.02; p=0.90), with attack rate reduction from baseline in both groups. Subgroups meeting a clinical definition of HAE [known mutations (n=5) or family history and unknown mutations (n=13)] showed positive attack rate reduction trend with lanadelumab versus placebo. Angioedema attack rate reduction with lanadelumab was observed in CASPIAN OLE. In both studies, all treatment-related TEAEs were non-serious, and most were non-severe; most frequent treatment-related TEAEs were similar to those previously reported in lanadelumab clinical trials.

**Conclusion:**

In patients with non-histaminergic nC1INH angioedema, lanadelumab safety was consistent with previous studies; efficacy remained inconclusive due to unmet CASPIAN primary endpoint. Overall results suggest potential clinical benefit in symptom control.

**Clinical trial registration:**

https://www.clinicaltrials.gov/, identifiers NCT04206605, NCT04444895.

## Graphical Abstract

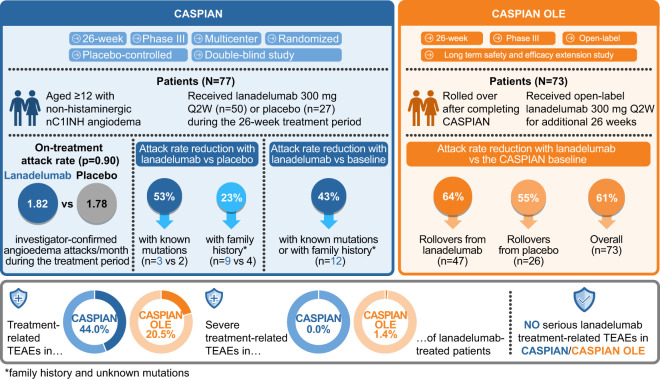

## 1 Introduction

Angioedema conditions are characterized by subcutaneous and/or submucosal swelling affecting the face, mouth, lips, extremities, gastrointestinal tract, genitalia, and/or upper airway (1, 2). Angioedema can be histaminergic (mast cell-mediated) or non-histaminergic (primarily bradykinin-mediated) (1–3).

Non-histaminergic angioedema may be hereditary or acquired and may present with C1 inhibitor (C1INH) deficiency or normal C1INH (nC1INH) (1, 4). Patients with hereditary angioedema (HAE) may present with quantitative deficiency (HAE Type I) or dysfunction (HAE Type II) of C1INH, or have nC1INH (HAE-nC1INH, previously referred to as HAE Type III) (5, 6).

HAE-nC1INH is a rare condition with an estimated prevalence of 0.37 per 100,000 people reported in the United States (7). Known mutations associated with HAE-nC1INH include mutations in coagulation factor XII (*F12*), plasminogen (*PLG*), angiopoietin-1 (*ANGPT1*), kininogen-1 (*KNG1*), myoferlin (*MYOF*), heparan sulfate 3-O-sulfotransferase 6 (*HS3ST6*), carboxypeptidase N (*CPN1*), and disabled homolog 2-interacting protein (*DAB2IP*) genes, although some causative genes are yet unknown (5, 8, 9). Diagnosis of HAE-nC1INH has been based on evaluation by an expert clinician based on clinical presentation and either known mutation associated with disease or family history of angioedema and a lack of efficacy of chronic high-dose antihistamine therapy (6, 10). More recently, it has also been recommended to consider a lack of response to omalizumab treatment for diagnosis of non-histaminergic angioedema, including HAE-nC1INH (4).

Other types of non-histaminergic nC1INH angioedema include acquired angioedema due to angiotensin-converting enzyme inhibitors and idiopathic non-histaminergic angioedema (1, 4). Diagnosis of idiopathic non-histaminergic angioedema is considered in patients with no family history but with recurrent angioedema despite the use of high-dose antihistamines and, more recently, omalizumab, once all other angioedema causes have been ruled out (1, 4).

Current recommendations on treatments for the prevention of angioedema attacks in patients with non-histaminergic nC1INH angioedema are based on open-label studies, case reports, or expert consensus due to the lack of data from randomized controlled trials (3, 11, 12).

Lanadelumab is a fully human monoclonal antibody inhibiting plasma kallikrein (pKal) approved for the prevention of HAE attacks in patients with HAE aged ≥12 years in multiple countries and regions, including the United States, the European Union, Canada, and China (13–17). In the United States and the European Union, lanadelumab indication was extended to include patients with HAE aged ≥2 years to 12 years in 2023 (15, 16). Lanadelumab is a highly potent and specific inhibitor of pKal; pKal inhibition by lanadelumab subsequently results in decreased levels of bradykinin (13). The effectiveness and safety of lanadelumab in preventing HAE attacks in patients with HAE Type I/II were investigated in the pivotal phase III HELP Study (NCT02586805) and the phase III HELP open-label extension (OLE; NCT02741596) Study (18, 19). We hypothesized that, based on the mechanism of action and known lanadelumab efficacy and safety, prophylactic treatment with lanadelumab may also be beneficial in other forms of angioedema such as non-histaminergic nC1INH angioedema.

Here, we report the results of the phase III, multicenter, randomized, placebo-controlled, double-blind CASPIAN Study (NCT04206605), which evaluated the efficacy and safety of lanadelumab in patients with non-histaminergic nC1INH angioedema, and its open-label, long-term safety and efficacy extension study (CASPIAN OLE; NCT04444895).

## 2 Methods

### 2.1 Study designs

CASPIAN and CASPIAN OLE were conducted in accordance with International Council for Harmonisation of Good Clinical Practice E6 guideline and the ethical principles described in the Declaration of Helsinki and other applicable local ethical and legal requirements. Institutional Review Board/Independent Ethics Committee approvals were obtained prior to each site initiation.

#### 2.1.1 CASPIAN

CASPIAN was a phase III, multicenter, randomized, placebo-controlled, double-blind study to evaluate the efficacy and safety of lanadelumab in preventing acute attacks of non-histaminergic angioedema in patients with nC1INH. The study was conducted at 34 sites in 10 countries, namely, Canada, France, Germany, Hungary, Italy, Japan, the Netherlands, Poland, Spain, and the United States, between 4 May 2020 and 20 October 2022.

**Figure 1:**
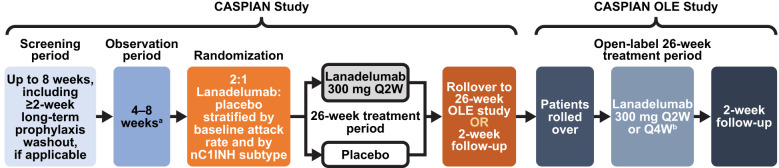
Study design of CASPIAN and CASPIAN OLE studies. ^a^Confirmation of negative response to high-dose antihistamines. ^b^Patients may consider switching to lanadelumab 300 mg Q4W if they have been well controlled (e.g., attack-free) for 26 consecutive weeks across CASPIAN and CASPIAN OLE. nC1INH, normal C1 inhibitor; OLE, open-label extension; Q2W, every 2 weeks; Q4W, every 4 weeks.

CASPIAN comprised a screening period of up to 8 weeks and an observation period (at least 4 weeks and up to 8 weeks) to identify patients eligible for randomization into the 26-week, double-blind treatment period (**Figure 1**). Patients who met all eligibility criteria at screening entered the observation period to determine the baseline angioedema attack rate and further confirm eligibility (additional details in *Study populations* below). Patients who had been on any long-term prophylaxis (e.g., C1INH, androgens, or antifibrinolytics) were required to undergo a minimum 2-week washout prior to the observation period. Patients who had a baseline angioedema attack rate of at least one investigator-confirmed angioedema attack per 4 weeks during the observation period while being treated with chronic high-dose antihistamines were randomized to treatment. For adult patients (aged ≥18 years), acute angioedema attacks during the observation and treatment periods were managed with icatibant. For adolescent patients (aged 12 years to <18 years), standard-of-care therapy per local protocols was provided for acute angioedema attacks.

Blood samples for genetic testing were collected from all patients during the screening period and predose on Day 0. Blood samples were tested in a central laboratory (ALMAC, Craigavon, UK) using a panel of six genetic variants in four genes, known, at the time, to be associated with HAE-nC1INH (*ANGPT1*_c.807G>T, *F12*_c.983C>A, *F12*_c.983C>G, *F12*_c.971_10181 + 24del72, *KNG1*_c.1136T>A, and *PLG*_c.988A>G).

Patients were randomized 1:2 to subcutaneous placebo every 2 weeks (Q2W) or subcutaneous lanadelumab 300 mg Q2W for 26 weeks using interactive response technology. Randomization was stratified by baseline angioedema attack rate (1 to <2 attacks/4 weeks and ≥2 attacks/4 weeks) and by nC1INH subtype [with known mutations (*F12, PLG, ANGPT1*, or *KNG1* genes associated with nC1INH angioedema); with family history (a first-degree relative) of angioedema and unknown mutations; or with idiopathic non-histaminergic angioedema]. All study site personnel, patients, healthcare providers, and the sponsor were blinded to study treatment until the study was complete.

Upon completion of the blinded treatment period (Day 182), patients were either discharged from CASPIAN and rolled over into the CASPIAN OLE Study (rollovers) or entered a 2-week safety follow-up period prior to discharge.

#### 2.1.2 CASPIAN OLE

CASPIAN OLE was a 26-week, phase III, open-label, long-term safety and efficacy extension study. Patients who agreed to continue into the OLE completed all study assessments for CASPIAN on the Week 26 study visit/Day 182 and rolled over to CASPIAN OLE at that study visit (**Figure 1**). The results of the CASPIAN Day 182 assessments were used as the pre-dose results for Day 0 of CASPIAN OLE.

After the first open-label dose of lanadelumab, patients received open-label subcutaneous lanadelumab 300 mg Q2W or every 4 weeks (Q4W) if attacks were well-controlled for 26 consecutive weeks across CASPIAN and CASPIAN OLE. Patients received open-label lanadelumab for 26 weeks/182 days after their first open-label dose, with a maximum of 13 doses administered during this period. After the completion of the treatment period, all patients underwent safety evaluations during a 2-week follow-up period.

### 2.2 Study populations

#### 2.2.1 CASPIAN

Male and female patients aged ≥12 years with a documented clinical history of recurrent attacks of angioedema in the absence of wheals/urticaria, ≥1 angioedema attack per 4 weeks prior to screening, and an investigator-confirmed diagnosis of non-histaminergic nC1INH angioedema were eligible for enrollment into CASPIAN.

Patients were required to have diagnostic testing results obtained during screening from a sponsor-approved central laboratory that confirmed C1INH function ≥50% of normal and complement component 4 (C4) level not below the normal range and clinical history demonstrating a lack of response to daily high-dose preventative antihistamine treatment (cetirizine 40 mg/day or equivalent high-dose second-generation antihistamine, as determined by the judgement of the investigator). Additionally, patients with a recorded response to omalizumab, corticosteroids, epinephrine, or leukotriene receptor antagonists in their past medical history were not eligible to enroll. Confirmation of patient non-response to high-dose antihistamine treatment was required to be confirmed during the observation period, with ≥1 angioedema attack per 4 weeks while on chronic high-dose antihistamine treatment and no significant difference (as assessed by the investigator) from the historic attack rate without high-dose antihistamine treatment. Patients aged ≥18 years had to be willing to use icatibant as a rescue treatment for angioedema attacks that occurred during the observation and treatment periods. Patients who, in the past medical history/screening, had no response to icatibant for acute angioedema attacks, as judged by the investigator, or no improvement or worsened attack severity 2 h after icatibant treatment during the observation period were not eligible to enroll.

Patients with a diagnosis of HAE Type I/II or recurrent angioedema associated with urticaria were not eligible. Patients were not permitted to join the study if they used short-term prophylaxis for HAE within 7 days or long-term prophylaxis for HAE within 2 weeks prior to entering the observation period or had exposure to investigational drugs or estrogen-containing medications within 4 weeks prior to screening, exposure to angiotensin-converting enzyme inhibitors or rituximab within 6 months prior to screening, or any exposure to prophylactic pKal inhibitors prior to screening.

All patients or legal guardians of patients provided informed consent to enter CASPIAN. Full inclusion and exclusion criteria for CASPIAN are listed in **Supplementary Table S1**.

#### 2.2.2 CASPIAN OLE

Patients who completed the double-blind treatment period (Day 182) of CASPIAN without reporting a clinically significant treatment-emergent adverse event (TEAE) that would preclude subsequent exposure to lanadelumab and who provided informed consent to roll over into CASPIAN OLE were eligible for inclusion. Full inclusion and exclusion criteria for CASPIAN OLE are listed in **Supplementary Table S2**.

### 2.3 Outcome measures and assessments

#### 2.3.1 CASPIAN

The primary efficacy endpoint was the number of investigator-confirmed attacks during the treatment period (Days 0–182).

Secondary efficacy endpoints included the number of patients achieving attack-free status during the treatment period and lanadelumab steady state (Days 70–182), the number of investigator-confirmed moderate or severe angioedema attacks during the treatment period and lanadelumab steady state, and the number of angioedema attacks during the lanadelumab steady state.

Other endpoints included lanadelumab pharmacokinetics (PK), pharmacodynamics (PD), immunogenicity, and health-related quality of life (HRQoL). Lanadelumab PK was evaluated by the plasma concentrations of lanadelumab. Lanadelumab PD was evaluated by the pKal activity and plasma cleaved high-molecular-weight kininogen (cHMWK) activity. Immunogenicity was evaluated by the presence of neutralizing or non-neutralizing anti-drug antibodies (ADAs) in plasma. HRQoL was evaluated by the Angioedema Quality of Life (AE-QoL) questionnaire, which contains 17 items in four domains (Functioning, Fatigue/Mood, Fears/Shame, and Nutrition) (20). AE-QoL domain and total scores are transformed to a scale of 0–100; higher AE-QoL scores reflect greater HRQoL impairment.

Safety endpoints included TEAEs, adverse events of special interest (AESIs; in the CASPIAN Study, prespecified AESIs were hypersensitivity reactions), and serious adverse events (SAEs). Although angioedema attacks also were captured as adverse events, they were summarized only in the efficacy analysis. TEAEs were classified by System Organ Class and Preferred Term using version 25.0 of Medical Dictionary for Regulatory Activities (MedDRA).

#### 2.3.2 CASPIAN OLE

The primary outcome was long-term safety of lanadelumab in patients with non-histaminergic nC1INH angioedema as evaluated by adverse events (AEs), SAEs, AESIs, clinical laboratory parameters, vital signs, and 12-lead electrocardiogram (ECG). Secondary efficacy endpoints included the number of investigator-confirmed angioedema attacks during the treatment period.

For PK/PD evaluations, plasma concentrations of lanadelumab, cHMWK levels, and pKal activity were measured. For immunogenicity evaluations, the presence of neutralizing or non-neutralizing ADAs was evaluated. HRQoL was assessed using the AE-QoL questionnaire.

### 2.4 Sample size

Approximately 75 patients with non-histaminergic nC1INH angioedema were planned to be included in the CASPIAN Study. Assuming a treatment effect of ≥60% reduction in the investigator-confirmed attack rate compared with placebo, which was based on the results from the HELP Study, and an attack rate of one attack per 4 weeks during the analysis period, a sample size of 75 patients would provide ≥85% power at one-sided α=0.025.

No formal sample size calculation was performed for CASPIAN OLE.

### 2.5 Statistical analysis

All statistical analyses were performed using SAS^®^ Version 9.4 (SAS Institute, Cary, NC, USA).

#### 2.5.1 CASPIAN

Efficacy analyses were conducted using the full analysis set (FAS), which included all randomized patients who received any exposure to the investigational product during the treatment period, analyzed according to the randomized treatment assignment regardless of the treatment actually received.

The primary efficacy endpoint was analyzed using a generalized linear model for count data assuming a Poisson distribution with a log link function and Pearson chi-square scaling of standard errors to account for potential overdispersion. The model included fixed effects for treatment group (categorical), normalized baseline attack rate (continuous), and stratification factor of subtype (categorical). The logarithm of time in days that each patient was observed during the treatment period was used as an offset variable in the model. From this model, the least squares mean attack rates with 95% confidence interval (CI) and the mean attack rate ratios relative to the placebo group with corresponding 95% CI were estimated. These estimates were reported as mean event rates per month, where month was defined as 4 weeks or 28 days, by transforming the estimates using the exponential function and scaling by the unit of time.

Prespecified subgroup analyses of the primary efficacy endpoint based on nC1INH subtypes (with known mutations, with family history and unknown mutations, and with idiopathic non-histaminergic angioedema) were also conducted. A *post-hoc* analysis of the primary efficacy endpoint in the combined subgroup of patients meeting a clinical definition of HAE (patients with known mutations and patients with family history and unknown mutations) was also performed.

A sensitivity analysis on the primary efficacy endpoint was also conducted in the modified FAS population, which excluded patients who did not meet eligibility criteria but were still randomized.

Safety analyses were conducted using the safety analysis set, which included all patients who received any exposure to the study treatment analyzed according to the treatment actually received regardless of randomized treatment assignment.

PK and PD outcomes were assessed in the respective PK and PD analysis sets, defined as patients who received at least one dose of the study treatment and had at least one post-dose PK or PD concentration value, respectively. No formal statistical hypotheses were tested for PK/PD. PK/PD parameters were summarized using descriptive statistics.

HRQoL was analyzed in the safety analysis set by tabulating AE-QoL responses for each of the 17 AE-QoL questions for each treatment group.

#### 2.5.2 CASPIAN OLE

No formal hypothesis testing was performed in CASPIAN OLE.

Safety analyses were conducted in the safety full analysis set (SFAS), which included all patients who received open-label lanadelumab after entering CASPIAN OLE and were summarized using descriptive statistics.

The efficacy endpoint, the number of investigator-confirmed angioedema attacks during the treatment period (Days 0–182), expressed as a monthly angioedema attack rate, was analyzed in the SFAS. The attack rate in the observation period of CASPIAN served as the baseline for CASPIAN OLE. The baseline attack rate, treatment period attack rate, and change from baseline in the attack rate during the treatment period were summarized for the overall population and by nC1INH subtype.

An integrated *post-hoc* analysis examined the mean monthly attack rate from baseline of CASPIAN and during the treatment periods of CASPIAN and CASPIAN OLE for patients who rolled over to CASPIAN OLE after placebo treatment in CASPIAN (rollovers from placebo) and patients who rolled over to CASPIAN OLE after lanadelumab treatment in CASPIAN (rollovers from lanadelumab). The difference in attack rate between the CASPIAN and CASPIAN OLE treatment periods was compared using paired t-test and Wilcoxon signed-rank test for each treatment group.

All summaries and analyses of the PK and PD data were based on the PK set and PD set, respectively; these analysis sets included all subjects in the SFAS who had at least one evaluable post-dose PK (PK set) or PD (PD set) concentration value. The plasma lanadelumab concentrations, cHMWK levels, and pKal activity were summarized using descriptive statistics.

HRQoL was assessed by calculating the AE-QoL total and domain scores.

## 3 Results

### 3.1 CASPIAN

#### 3.1.1 Patient disposition

Of the 119 patients who were screened, 48 were screen failures. Most frequent reasons for failing the screening included not meeting the inclusion criteria related to either investigator-confirmed diagnosis of non-histaminergic nC1INH angioedema or angioedema attack rate of at least one attack per 4 weeks. Overall, 77 patients [including six who did not meet eligibility criteria and were screen failures but participated in and completed the study (patients not meeting eligibility criteria were identified only after the patients completed the study)] comprised the FAS; 50 were randomized to lanadelumab 300 mg Q2W and 27 to placebo. Of the 27 patients who received placebo, 25 (92.6%) completed the treatment period, and 26 (96.3%) completed the study; one patient who did not complete the study withdrew. Of the 50 patients who received lanadelumab, 48 (96.0%) completed the treatment period, and 49 (98.0%) completed the study; one patient who did not complete the study discontinued due to an AE. Patient flow diagram is presented in **Supplementary Figure S1A**.

#### 3.1.2 Patient demographics and baseline characteristics

Patient age (mean ± SD) was 42.8 ± 12.9 years, and most patients were women (80.5%), White (88.3%), and not Hispanic or Latino (87.0%). No adolescent patients aged 12 years to <18 years were enrolled in CASPIAN. The age range of enrolled patients was 18–72 years.

By nC1INH subtypes, five patients had known mutations (four with *PLG* mutations and one with *F12* mutation; three randomized to lanadelumab and two to placebo), 13 had family history and unknown mutations (nine randomized to lanadelumab and four to placebo), and 59 had idiopathic non-histaminergic angioedema (38 randomized to lanadelumab and 21 to placebo).

Overall, demographics and baseline characteristics were comparable between the lanadelumab and placebo groups (**Table 1**), except historical attack rate, which was numerically higher in the lanadelumab group compared with the placebo group.

**Table 1:** Baseline demographics and clinical characteristics.

Characteristic	Placebo (n=27)	Lanadelumab 300 mg Q2W (n=50)	Total (N=77)
Age, years, mean ± SD	43.8 ± 10.8	42.3 ± 14.1	42.8 ± 12.9
Sex, n (%)			
Female	19 (70.4)	43 (86.0)	62 (80.5)
Male	8 (29.6)	7 (14.0)	15 (19.5)
Race, n (%)			
White	24 (88.9)	44 (88.0)	68 (88.3)
Black or African American	0	4 (8.0)	4 (5.2)
Asian	2 (7.4)	2 (4.0)	4 (5.2)
Other	1 (3.7)	0	1 (1.3)
Ethnicity, n (%)			
Not Hispanic or Latino	24 (88.9)	43 (86.0)	67 (87.0)
Hispanic or Latino	2 (7.4)	7 (14.0)	9 (11.7)
Not reported	1 (3.7)	0	1 (1.3)
Weight, kg, mean ± SD	82.1 ± 22.7	82.0 ± 25.2	82.1 ± 24.2
BMI, kg/m^2^, mean ± SD	28.9 ± 7.4	29.8 ± 9.1	29.4 ± 8.5
Historical attack rate (attacks/month),^a^ mean ± SD	3.9 ± 4.4	5.3 ± 5.5	4.8 ± 5.1
Normal C1INH subtype, n (%)			
With known mutations	2 (7.4)	3 (6.0)	5 (6.5)
With family history and unknown mutations	4 (14.8)	9 (18.0)	13 (16.9)
Idiopathic	21 (77.8)	38 (76.0)	59 (76.6)

BMI, body mass index; nC1INH, normal C1 inhibitor; Q2W, every 2 weeks; SD, standard deviation.

a Calculated from the number of attacks in the last 3 months.

There were no notable differences between the treatment groups in biomarker test results at screening. The mean ± SD C1INH function at screening was 152.3 ± 38.40% of normal in the lanadelumab group and 150.0 ± 41.43% of normal in the placebo group. The mean ± SD C4 concentration at screening was 28.2 ± 7.0 mg/dL in the lanadelumab group and 27.3 ± 6.5 mg/dL in the placebo group.

#### 3.1.3 Adherence to treatment

All 77 (100%) patients received ≥80% of the planned doses. The patients received a total of mean ± SD of 12.7 ± 1.6 doses during the treatment period, which corresponded to a mean ± SD of 99.7 ± 1.5% of the planned doses received. There were no notable differences in the adherence to treatment between the lanadelumab and placebo groups.

#### 3.1.4 Primary efficacy endpoint

The mean ± SD rate of investigator-confirmed angioedema attacks/month during the observation and treatment periods decreased from 3.93 ± 2.89 to 2.17 ± 2.06 attacks/month for patients who received lanadelumab (mean ± SD change from baseline, −1.76 ± 1.99) and from 2.78 ± 1.55 to 1.63 ± 1.36 attacks/month for patients who received placebo (mean ± SD change from baseline, −1.15 ± 1.91) (**Table 2**).

**Table 2:** Investigator-confirmed angioedema attacks during the treatment period in CASPIAN by study treatment.

Overall	Placebo (n=27)	Lanadelumab 300 mg Q2W (n=50)
Investigator-confirmed angioedema attacks during the treatment period (Days 0–182)		
Observation period angioedema attack rate, attacks/month, mean ± SD	2.78 ± 1.55	3.93 ± 2.89
Treatment period angioedema attack rate, attacks/month, mean ± SD	1.63 ± 1.36	2.17 ± 2.06
Angioedema attack rate model-based analysis		
Estimated LS rate, attacks/month, mean (95% CI)	1.78 (1.24–2.55)	1.82 (1.37–2.42)
Rate ratio relative to placebo (95% CI)		1.02 (0.71–1.47)
p-value		0.90
Percent change in mean rate vs. placebo (95% CI)		2.38 (−28.71 to 47.03)

Data were analyzed in the full analysis set.

CI, confidence interval; LS, least squares; Q2W, every 2 weeks; SD, standard deviation.

The primary efficacy endpoint of model-based angioedema attack rate during the treatment period was not significantly different between the lanadelumab and placebo groups (**Table 2**). The estimated mean angioedema attack rate was 1.82 (95% CI, 1.37–2.42) attacks/month for patients who received lanadelumab and 1.78 (95% CI, 1.24–2.55) attacks/month for those who received placebo (the rate ratio relative to placebo was 1.02; 95% CI, 0.71–1.47; p=0.90). A sensitivity analysis showed no notable difference in the investigator-confirmed angioedema attack rate between the FAS and a modified FAS that excluded the six patients who did not meet eligibility criteria (data not shown).

In the prespecified subgroup analysis of the investigator-confirmed angioedema attacks during the treatment period based on the nC1INH subtype, no significant differences with lanadelumab versus placebo were observed in any of the three subgroups (**Table 3**).

**Table 3:** Investigator-confirmed angioedema attacks during the treatment period in CASPIAN and by study treatment and by nC1INH subgroup.

Non-histaminergic nC1INH angioedema with known mutations	Placebo (n=2)	Lanadelumab 300 mg Q2W (n=3)
Investigator-confirmed angioedema attacks during the treatment period (Days 0–182)		
Observation period angioedema attack rate, attacks/month, mean ± SD	1.25 ± 0.35	4.33 ± 2.89
Treatment period angioedema attack rate, attacks/month, mean ± SD	2.04 ± 0.08	1.58 ± 1.06
Angioedema attack rate model-based analysis		
Estimated LS rate, attacks/month, mean (95% CI)	2.66 (1.16–6.11)	1.24 (0.55–2.82)
Rate ratio relative to placebo (95% CI)		0.47 (0.11–1.91)
p-value		0.29
Percent change in mean rate vs. placebo (95% CI)		−53.29 (−88.58 to 91.07)
Non-histaminergic nC1INH angioedema with family history and unknown mutations	Placebo (n=4)	Lanadelumab 300 mg Q2W (n=9)
Investigator-confirmed angioedema attacks during the treatment period (Days 0–182)		
Observation period angioedema attack rate, attacks/month, mean ± SD	2.25 ± 0.96	2.89 ± 1.75
Treatment period angioedema attack rate, attacks/month, mean ± SD	1.92 ± 1.34	1.66 ± 1.77
Angioedema attack rate model-based analysis		
Estimated LS rate, attacks/month, mean (95% CI)	1.99 (0.80–4.91)	1.53 (0.77–3.06)
Rate ratio relative to placebo (95% CI)		0.77 (0.24–2.47)
p-value		0.66
Percent change in mean rate vs. placebo (95% CI)		−23.00 (−75.98 to 146.78)
Idiopathic non-histaminergic angioedema	Placebo (n=21)	Lanadelumab 300 mg Q2W (n=38)
Investigator-confirmed angioedema attacks during the treatment period (Days 0–182)		
Observation period angioedema attack rate, attacks/month, mean ± SD	3.03 ± 1.62	4.14 ± 3.10
Treatment period angioedema attack rate, attacks/month, mean ± SD	1.53 ± 1.44	2.34 ± 2.18
Angioedema attack rate model-based analysis		
Estimated LS rate, attacks/month, mean (95% CI)	1.65 (1.19–2.30)	1.92 (1.53–2.41)
Rate ratio relative to placebo (95% CI)		1.16 (0.77–1.74)
p-value		0.47
Percent change in mean rate vs. placebo (95% CI)		16.00 (−22.57 to 73.77)

Data were analyzed in the full analysis set.

CI, confidence interval; LS, least squares; Q2W, every 2 weeks; SD, standard deviation.

A *post-hoc* analysis combined patients with known mutations and patients with known family history and unknown mutations (**Table 4**). In this analysis, the mean ± SD rate of investigator-confirmed angioedema attacks in the 12 patients treated with lanadelumab decreased from 3.25 ± 2.04 attacks/month during the observation period to 1.64 ± 1.58 attacks/month during the treatment period, which represented a mean ± SD change of −1.61 ± 2.09 attacks/month and a mean ± SD percent change of −42.63 ± 46.00 (95% CI, −71.9 to −13.4) from baseline during the treatment period. In the six patients receiving placebo, there was a minimal change in the mean ± SD rate of investigator-confirmed angioedema attacks from the observation period to the treatment period (from 1.92 ± 0.92 to 1.96 ± 1.04 attacks/month). The model-estimated mean angioedema attack rate during treatment period was 1.46 (95% CI, 0.80–2.66) attacks/month in patients from the lanadelumab group and 2.12 (95% CI, 1.06–4.20) attacks/month in patients from the placebo group. The angioedema attack rate was numerically lower with lanadelumab versus placebo (rate ratio, 0.69; p=0.43).

**Table 4:** *Post-hoc* analysis of investigator-confirmed angioedema attacks during the treatment period in CASPIAN in a combined subgroup of patients with non-histaminergic nC1INH angioedema with known mutations or with family history and unknown mutations.

	Placebo (n=6)	Lanadelumab 300 mg Q2W (n=12)
Investigator-confirmed angioedema attacks during the treatment period (Days 0–182)		
Observation period angioedema attack rate, attacks/month, mean ± SD	1.92 ± 0.92	3.25 ± 2.04
Treatment period angioedema attack rate, attacks/month, mean ± SD	1.96 ± 1.04	1.64 ± 1.58
Angioedema attack rate model-based analysis		
Estimated LS rate, attacks/month, mean (95% CI)	2.12 (1.06–4.20)	1.46 (0.80–2.66)
Rate ratio relative to placebo (95% CI)		0.69 (0.28–1.73)
p-value		0.43
Percent change in mean rate vs. placebo (95% CI)		−30.93 (−72.36 to 72.62)

Data were analyzed in the full analysis set.

CI, confidence interval; LS, least squares; Q2W, every 2 weeks; SD, standard deviation.

As the statistical significance between treatment groups was not met for the primary endpoint, secondary efficacy endpoints are not reported.

#### 3.1.5 Pharmacokinetic and pharmacodynamic outcomes

The lanadelumab steady-state concentration was achieved by the Day 56 visit for the majority of patients (the mean ± SD pre-dose concentration on the Day 56 visit was 20.7 ± 8.40 μg/mL) (**Supplementary Figure S2**).

The mean ± SD pre-dose concentrations at visits on Days 84, 112, 140, and 168 ranged from 21.4 ± 11.1 μg/mL to 22.9 ± 10.0 μg/mL. Lanadelumab plasma concentrations were similar across the different nC1INH subgroups.

On average, patients treated with lanadelumab achieved a steady-state pKal inhibition of approximately 50% by the Day 56 visit (**Supplementary Figure S3**). This inhibition was sustained and consistent with the observed PK profile over time. The mean percent change from baseline levels of pKal for patients who received placebo ranged from −10.7% to +6% throughout the treatment period.

cHMWK results showed variability in the percent change from baseline over visit in patients who received placebo compared with those treated with lanadelumab (**Supplementary Figure S4**). There was a trend in the reduction in cHMWK activity in the lanadelumab group compared with placebo.

For both pKal and cHMWK activity, inhibition was numerically higher in patients with nC1INH with known mutations or family history compared with patients with idiopathic nC1INH.

#### 3.1.6 HRQoL

Overall, there was improvement in all AE-QoL items in both treatment groups at the end of the study. A general comparison of the results by visit showed that improvements were continually noted across all dimensions as the study progressed. The largest improvements were often noted between baseline (Day 0) and end of treatment (Day 182). The results showed greater improvement in lanadelumab-treated patients compared with those receiving placebo.

#### 3.1.7 Lanadelumab exposure

Patients randomized to lanadelumab received a mean ± SD number of 12.7 ± 1.7 doses of treatment during the study. The mean ± SD total dose was 3,802.5 ± 512.1 mg, and the mean ± SD duration of exposure to lanadelumab was 5.9 ± 0.9 months.

#### 3.1.8 Safety and immunogenicity

During the treatment period, 46 of 50 (92.0%) patients in the lanadelumab group reported 296 TEAEs, and 23 of 27 (85.2%) patients from the placebo group reported 138 TEAEs (**Table 5**). In the lanadelumab group, the most frequently reported TEAEs by MedDRA Preferred Term included injection site pain [61 events in 15 (30.0%) patients], arthralgia [eight events in seven (14.0%) patients], and headache [13 events in six (12.0%) patients].

**Table 5:** TEAEs in the CASPIAN Study by treatment group.

	Placebo (n=27)		Lanadelumab 300 mg Q2W (n=50)	
	Patients, n (%)	Events, n	Patients, n (%)	Events, n
TEAEs	23 (85.2)	138	46 (92.0)	296
TEAEs occurring in ≥10% of patients in either treatment group				
Injection site pain	7 (25.9)	9	15 (30.0)	61
Arthralgia	2 (7.4)	2	7 (14.0)	8
Headache	6 (22.2)	13	6 (12.0)	13
Nausea	3 (11.1)	8	4 (8.0)	4
COVID-19 infection	4 (14.8)	4	3 (6.0)	3
Injection site erythema	3 (11.1)	6	3 (6.0)	22
Upper respiratory tract infection	3 (11.1)	3	3 (6.0)	5
Insomnia	3 (11.1)	3	1 (2.0)	1
Treatment-related TEAEs	12 (44.4)	43	22 (44.0)	125
Serious TEAEs	0	0	2 (4.0)	2
Treatment-related serious TEAEs	0	0	0	0
Severe TEAEs	0	0	3 (6.0)	8
Treatment-related severe TEAEs	0	0	0	0
AESIs	0	0	1 (2.0)	1
Deaths due to TEAEs	0	0	0	0
Study discontinuations due to TEAEs	0	0	1 (2.0)	1

Data were analyzed in the safety analysis set.

AESI, adverse event of special interest; TEAE, treatment-emergent adverse event; Q2W, every 2 weeks; Q4W, every 4 weeks.

Treatment-related TEAEs were reported in 22 (44.0%) patients in the lanadelumab group (125 events) and in 12 (44.4%) patients in the placebo group (43 events). In the lanadelumab group, the most frequently reported treatment-related TEAEs by MedDRA Preferred Term included injection site pain [58 events in 14 (28.0%) patients], injection site erythema [21 events in two (4.0%) patients], fatigue [11 events in two (4.0%) patients], urticaria [three events in two (4.0%) patients], and headache [two events in two (4.0%) patients]. One patient treated with lanadelumab discontinued the study due to a TEAE of insomnia that was not considered related to treatment.

There were two SAEs in two patients treated with lanadelumab (lymphoedema and cellulitis staphylococcal); both were not considered related to treatment.

There was one investigator-reported AESI in the lanadelumab group [hypersensitivity (injection site rash)], which was assessed as treatment-related by the investigator and was moderate in severity.

Injection site reactions (ISRs) were reported by 19 (38.0%) patients treated with lanadelumab who reported 94 ISRs and 13 (48.1%) patients who received placebo and reported 29 ISRs. The majority of ISRs were mild in severity and resolved within 30 min of administration. There were no serious ISRs, hospitalizations, or study discontinuations due to ISRs.

Of the patients in the lanadelumab group, one (2.2%) was ADA positive at baseline and two (4.1%) were ADA positive at the end of treatment (Day 182); all patients had low titers (<20- to 160-fold) of neutralizing non-reactive antibodies.

### 3.2 CASPIAN OLE

#### 3.2.1 Patient population

Of the 75 patients who completed the CASPIAN Study, 73 entered CASPIAN OLE (26 rollovers from placebo and 47 rollovers from lanadelumab). All patients received lanadelumab Q2W in CASPIAN OLE; two switched to lanadelumab 300 mg Q4W during the study, with one of these switching back to the Q2W regimen. A total of 64 (87.7%) patients completed the treatment period and the study, and nine (12.3%) patients discontinued (five patients withdrew, two patients discontinued due to AEs and two were lost to follow-up). Patient flow is presented in **Supplementary Figure S1B**.

#### 3.2.2 Lanadelumab exposure

Overall, patients received a mean ± SD of 12.2 ± 2.1 doses of lanadelumab during CASPIAN OLE. The mean ± SD duration of exposure to lanadelumab was 5.7 ± 1.1 months.

#### 3.2.3 Safety and immunogenicity

During the treatment period of the CASPIAN OLE Study, 55 of 73 patients (75.3%) reported 295 TEAEs (**Table 6**). The most frequent TEAEs by MedDRA Preferred Term were COVID-19 [17 events in 17 (23.3%) patients], headache [23 events in 11 (15.1%) patients], and injection site pain [48 events in eight (11.0%) patients].

**Table 6:** TEAEs in the CASPIAN OLE Study.

CASPIAN OLE	Lanadelumab 300 mg Q2W/Q4W (n=73)	
	Patients, n (%)	Events, n
TEAEs	55 (75.3)	295
TEAEs occurring in ≥5% of patients		
COVID-19 infection	17 (23.3)	17
Headache	11 (15.1)	23
Injection site pain	8 (11.0)	48
Arthralgia	7 (9.6)	9
Nausea	6 (8.2)	8
Upper respiratory tract infection	6 (8.2)	7
Ligament sprain	4 (5.5)	5
Urticaria	4 (5.5)	4
Treatment-related TEAEs	15 (20.5)	94
Serious TEAEs	6 (8.2)	8
Treatment-related serious TEAEs	0	0
Severe TEAEs	10 (13.7)	13
Treatment-related severe TEAEs	1 (1.4)	1
AESIs	1 (1.4)	1
Deaths due to TEAEs	0	0
Study discontinuations due to TEAEs	1 (1.4)	2

Data were analyzed in the safety full analysis set.

AESI, adverse event of special interest; TEAE, treatment-emergent adverse event; Q2W, every 2 weeks; Q4W, every 4 weeks.

Treatment-related TEAEs were reported in 15 (20.5%) patients (94 events). Treatment-related TEAEs reported in ≥2 patients included injection site pain [47 events in eight (11.0%) patients], headache [five events in two (2.7%) patients], injection site swelling [four events in two (2.7%) patients], and urticaria [two events in two (2.7%) patients]. Two patients discontinued the study due to treatment-related TEAEs (peripheral edema, which was considered mild in severity, and nausea and dizziness, which were considered moderate in severity). Overall, eight SAEs were reported in six (8.2%) patients. All SAEs required hospitalization, and none were considered related to study treatment. No deaths were reported during the study. No hypersensitivity AESIs were reported during the follow-up. One (1.4%) patient who was a rollover from lanadelumab had an investigator-reported AESI of oral herpes, which was mild in severity and not considered related to treatment by the investigator. All ISRs were mild in severity; there were no serious ISRs and no study discontinuations due to ISRs.

No clinically meaningful safety findings were identified in terms of clinical laboratory tests, vital signs, or ECG. At Day 0 of the CASPIAN OLE Study, two (2.8%) patients who were rollovers from lanadelumab were ADA positive. At Day 182, four (5.8%) patients (two rollovers each from lanadelumab and placebo) were ADA positive. Only one patient (rollover from placebo) had a reactive neutralizing ADA result at Day 140; the rest were non-reactive neutralizing ADAs.

#### 3.2.4 Efficacy outcomes

Lanadelumab reduced the mean ± SD angioedema attack rate over the 26-week treatment period in CASPIAN OLE from an attack rate of 3.6 ± 2.58 attacks/month (baseline attack rate during CASPIAN observation period) to 1.3 ± 1.46 attacks/month, which represented a mean ± SD percent change of −60.8 ± 44.84 (**Table 7**). The respective mean ± SD percent change in monthly attack rate by lanadelumab treatment was −55.1 ± 59.66 and −64.1 ± 34.40 for rollovers from placebo and lanadelumab groups, respectively. The attack rate reduction from baseline was consistently observed for all three nC1INH subtypes (**Table 7**).

**Table 7:** Investigator-confirmed angioedema attacks in the CASPIAN OLE Study by previous treatment group in the CASPIAN Study and by nC1INH subtype.

	Lanadelumab 300 mg Q2W		
	By previous treatment group in CASPIAN		
	Rollovers from placebo (n=26)	Rollovers from lanadelumab (n=47)	Overall (N=73)
Baseline observation period angioedema attack rate, attacks/month, mean ± SD	2.8 ± 1.58	4.0 ± 2.92	3.6 ± 2.58
Treatment period (Q2W) angioedema attack rate, attacks/month, mean ± SD	1.0 ± 1.05	1.5 ± 1.62	1.3 ± 1.46
Percent change from baseline attack rate, mean ± SD	−55.1 ± 59.66	−64.1 ± 34.40	−60.8 ± 44.84
	By nC1INH subtype		
	With known mutations (n=5)	With family history and unknown mutations (n=13)	Idiopathic nC1INH angioedema (n=55)
Baseline observation period angioedema attack rate, attacks/month, mean ± SD	3.1 ± 2.66	2.7 ± 1.53	3.8 ± 2.75
Treatment period (Q2W) angioedema attack rate, attacks/month, mean ± SD	1.8 ± 1.00	1.0 ± 1.35	1.3 ± 1.52
Percent change from baseline attack rate, mean ± SD	−1.5 ± 108.66	−58.2 ± 47.64	−66.9 ± 30.62

nC1INH, normal C1 inhibitor; Q2W, every 2 weeks; SD, standard deviation.

In the integrated *post-hoc* efficacy analysis, the monthly mean ± SD angioedema attack rate in rollovers from placebo decreased from 2.8 ± 1.58 attacks/month at baseline to 1.7 ± 1.36 attacks/month in CASPIAN and 1.0 ± 1.05 attacks/month in CASPIAN OLE, for a difference of −0.7 ± 0.83 between CASPIAN and CASPIAN OLE (p=0.0004 paired t-test and p=0.0001 Wilcoxon signed-rank test).

Rollovers from lanadelumab continued to have a decrease in monthly mean ± SD angioedema attack rate during CASPIAN OLE; the angioedema attack rate in these patients decreased from 4.0 ± 2.92 attacks/month at baseline to 2.3 ± 2.08 attacks/month in CASPIAN and to 1.5 ± 1.62 attacks/month in CASPIAN OLE, for a difference of −0.8 ± 1.29 between CASPIAN and CASPIAN OLE (p<0.0001 paired t-test and p<0.0001 Wilcoxon signed-rank test).

#### 3.2.5 PK and PD outcomes

The mean plasma concentration of lanadelumab was similar across all visits after lanadelumab 300 mg Q2W administration, with mean trough values ranging between 14.4 μg/mL and 21.1 μg/mL. On average, patients achieved a steady-state pKal inhibition of approximately 16%–20% across study visits. The mean cHMWK activity was consistent across all visits and ranged from 15%–17% across study visits.

#### 3.2.6 HRQoL

Overall, the AE-QoL results showed improvements in all items as the study progressed, and there were greater improvements for rollovers from lanadelumab compared with rollovers from placebo.

## 4 Discussion

Lanadelumab is a pKal inhibitor approved for long-term prophylaxis to prevent attacks of HAE in patients with HAE aged ≥2 years in the United States and European Union, or ≥12 years in several other countries and regions (14–17). Lanadelumab has been used to treat patients with HAE since its initial approval in 2018 (15, 16) and is recommended by international guidelines as one of the first-line options for long-term prophylaxis in patients with HAE Type I/II (20). The benefit–risk profile of lanadelumab for the management of HAE Type I/II is well established; however, the unmet medical need in patients with non-histaminergic angioedema with nC1INH remains high. Based on the mechanism of action of lanadelumab, we hypothesized that prophylactic treatment with lanadelumab may be beneficial in patients with non-histaminergic nC1INH angioedema and therefore initiated the study to investigate lanadelumab in this population. To our knowledge, this is the only randomized controlled study to date to investigate the effectiveness of a prophylactic agent in patients with non-histaminergic nC1INH angioedema in an interventional study.

CASPIAN was a global study conducted in 10 countries. The original study protocol was prepared in 2019 and followed the understanding of non-histaminergic nC1INH angioedema at that time. This included assessing all patients for six specific mutations in four genes known, at the time of the study initiation, to be associated with HAE-nC1INH (*F12*, *PLG*, *ANGPT1*, and *KNG1*). Since the start of the study, mutations in four additional genes (*MYOF*, *HS3ST6*, *CPN1*, and *DAB2IP*) and additional variants in *F12* and *ANGPT1* genes have been reported to be associated with HAE-nC1INH (5, 8, 9); however, patients were not screened for those mutations in CASPIAN. While it is possible that some patients with these additional mutations may have been misclassified, it is unlikely considering the low frequencies of mutations in *MYOF* and *HS3ST6* genes among patients with nC1INH angioedema (21).

According to the recent DANCE (definition, acronyms, nomenclature, and classification of angioedema) consensus, the CASPIAN and CASPIAN OLE studies included patients with heterogeneous angioedema types (22). The five patients from the subgroup with known mutations had either HAE due to mutations in *F12* gene (HAE-FXII) or HAE due to mutations in *PLG* gene (HAE-PLG), both of which are classified as bradykinin-mediated angioedema (AE-BK). The 13 patients from the subgroup with family history and unknown mutations can be classified as having hereditary angioedema of unknown etiology/mechanism (HAE-UNK), considering that family history is suggestive of a hereditary element even when the exact causative mutation is unknown. The 59 patients from the subgroup of idiopathic non-histaminergic angioedema can be classified as having angioedema of unknown etiology/mechanism (AE-UNK); however, some of these patients may have had mast cell-mediated angioedema (AE-MC) due to the complexity of obtaining correct diagnosis in this population, as discussed in detail below.

In this study, the primary endpoint did not show a significant reduction in the number of angioedema attacks with lanadelumab compared with placebo during the treatment period in patients with investigator-diagnosed non-histaminergic nC1INH angioedema. These results are in contrast to the results of the HELP Study, which demonstrated a significant HAE attack rate reduction with lanadelumab versus placebo in patients with HAE Type I/II (18). The number of patients with known mutations or with a family history of angioedema was small, which led to most patients (59/77; 76.6%) being classified to the idiopathic non-histaminergic angioedema subgroup. Therefore, results for the primary endpoint were driven by this population. In the idiopathic subgroup, patients treated with lanadelumab had a higher baseline attack rate versus those receiving placebo. Furthermore, patients from the idiopathic subgroup who received placebo experienced a marked reduction in the attack rate on treatment. Both of these factors may have contributed to the CASPIAN Study not reaching its primary endpoint. Numerical reduction in the attack rate with lanadelumab versus placebo was observed in subgroups of patients with known mutations and with family history and unknown mutations. However, due to the small sample size in each subgroup (five patients with known mutations and 13 patients with family history and unknown mutations), no definitive conclusions can be made.

Diagnosing the mechanism of angioedema in patients with normal levels of C1INH and absence of urticaria is challenging (1, 3, 6, 10). Guidelines for diagnosis of HAE-nC1INH include (i) a history of recurrent angioedema in the absence of concomitant hives or use of medication known to cause angioedema; (ii) documented normal or near normal C4, C1INH antigen, and C1INH function; and (iii) either a genetic variant associated with the disease or a family history of angioedema and documented lack of efficacy of chronic, high-dose antihistamine therapy (10). Using these definitions, the subgroup with known mutations and the subgroup with family history and unknown mutations in the CASPIAN Study can be considered to accurately represent patients with HAE-nC1INH. However, these clinical criteria have well-recognized limitations including unknown or imprecise family history, recognition of *de novo* cases in all hereditary conditions, and challenges in determining response to mast cell–targeted treatments (3, 23).

Diagnosis of non-histaminergic idiopathic angioedema is even more difficult and is based on exclusion of any other causes of angioedema in patients presenting with angioedema, nC1INH, absence of urticaria, and a lack of response to mast cell–targeted therapy (1). Furthermore, the understanding of pathophysiology underlying non-histaminergic idiopathic angioedema is still evolving. At the time when the CASPIAN Study protocol was developed, differentiation between histaminergic and non-histaminergic angioedema primarily considered response to high-dose antihistamines, which is reflected in CASPIAN inclusion criteria (24). Although patients with a known history of response to omalizumab, corticosteroids, epinephrine, or leukotriene receptor antagonists were excluded from CASPIAN, the only confirmation required during the observation period prior to enrollment was a lack of response to antihistamines. However, in recent years, expert opinion has evolved to recognize that a lack of response to high-dose antihistamines may be insufficient to exclude an underlying mast cell–mediated mechanism; for example, it is known that a subset of patients with chronic spontaneous urticaria do not achieve symptom control with high-dose antihistamine treatment (25). Thus, a lack of response to antihistamines does not conclusively confirm that the particular case of angioedema is bradykinin-mediated (1). In bradykinin-mediated angioedema, bradykinin may be formed both through and outside the kallikrein–kinin system. For example, in HAE-nC1INH with mutations in the *PLG* gene, altered plasminogen bypasses kallikrein and directly cleaves HMWK to produce bradykinin (26) Furthermore, HAE-nC1INH with mutations in the *MYOF*, *ANGPT-1*, and *HS3ST6* genes may involve vascular permeability factors beyond bradykinin (4, 27, 28). This further highlights the complexities of diagnosing different forms of nC1INH angioedema.

Improvement with acute icatibant treatment has also been suggested to support diagnosis of bradykinin-mediated (non-histaminergic) idiopathic angioedema (1); accordingly, patients with a lack of response to icatibant were not included in CASPIAN. Furthermore, response to omalizumab is increasingly considered when classifying angioedema as histaminergic or non-histaminergic, with response to either antihistamines or omalizumab cited as consistent with histaminergic (mast cell–mediated) angioedema in recent literature (4, 29). Additionally, approximately 10% of patients with chronic spontaneous urticaria, a mast cell–mediated condition for which treatment with omalizumab after insufficient response to antihistamines is recommended, have been reported to present only with angioedema and without wheals (25, 30); these patients may be misdiagnosed with non-histaminergic angioedema with nC1INH. Full diagnostic workup, including comprehensive genetic testing for HAE-nC1INH variants and testing for omalizumab response, would help to better identify the underlying pathophysiology in patients with nC1INH angioedema and select the most effective treatment for the individual patient. However, genetic testing for every patient may not be realistic in real-world clinical practice, outside of controlled studies. Similarly, access to testing for omalizumab response may be restricted by requirements for prior authorization, limited insurance coverage, and restricted reimbursement, at least in some countries, as these restrictions have been reported for access to omalizumab in patients with chronic urticaria (31, 32).

In CASPIAN OLE, investigator-confirmed angioedema attacks decreased during the treatment period versus the observation period, with the attack rate reduced by 60.8% overall and by 55.1% and 64.1% in patients who received placebo and lanadelumab in the CASPIAN Study, respectively. Reductions were observed in subgroups of patients with family history and idiopathic disease. Variance in reduction was observed in patients with known mutations; however, some of these patients had a low baseline attack rate, and the sample size was small. An exploratory analysis on attack rate reduction in CASPIAN OLE compared with CASPIAN showed further reductions in HAE attack rate in CASPIAN OLE, in rollovers from both the placebo and lanadelumab arms in CASPIAN, demonstrating a treatment effect of lanadelumab during the open-label treatment period.

Lanadelumab PK and PD results were consistent with known lanadelumab PK/PD profile (33). Plasma concentrations of lanadelumab reached steady state by Day 56 with no difference across the nC1INH subtypes. Patients in the lanadelumab group had a marked and sustained reduction in pKal activity; furthermore, an apparent trend of reduction in cHMWK activity was observed with lanadelumab versus placebo. These findings are consistent with the lanadelumab mechanism of action (13), suggesting that lanadelumab was active in patients with non-histaminergic nC1INH angioedema.

Improvements in HRQoL as measured by the AE-QoL were observed in both treatment arms in the CASPIAN Study. HRQoL improvements continued during the CASPIAN OLE Study. These findings are consistent with the results from the HELP and HELP OLE studies that reported HRQoL improvements with lanadelumab treatment in patients with HAE Type I/II (34, 35).

Lanadelumab safety was consistent with that previously reported in studies with patients in HAE Type I/II and similar between lanadelumab and placebo arms in CASPIAN. Similar to the HELP and HELP OLE studies in patients with HAE Type I/II, most lanadelumab TEAEs were non-serious and not severe (18, 19). Injection site TEAEs were among the most frequently reported TEAEs in the CASPIAN Study, consistent with the findings of the HELP and HELP OLE studies (18, 19).

The CASPIAN Study had several limitations. First, the study results may have been confounded by the high response in patients receiving placebo. Additionally, diagnosis of non-histaminergic (non-mast cell–mediated) idiopathic angioedema poses challenges, which may have resulted in recruitment of patients who were misdiagnosed with this condition. Although patients with a history of response to omalizumab were not eligible to enroll to CASPIAN, confirmation of a lack of response to omalizumab was not required during the observation period, which may have resulted in the enrollment of some patients who had histaminergic angioedema despite a lack of response to preventative high-dose antihistamines. Limitations in the largely clinical inclusion/exclusion criteria highlight the need for additional validated biomarkers to provide greater precision and confidence in the diagnosis of angioedema conditions not mediated by mast cells. Furthermore, there were few patients in subgroups with known mutations or family history and unknown mutations, who were considered to have HAE-nC1INH in the CASPIAN Study, likely due to rarity of HAE-nC1INH overall and the identified gene variants associated with HAE-nC1INH. This prevents any definitive conclusions about lanadelumab efficacy in HAE-nC1INH. Additionally, patients were screened only for mutations known to be associated with HAE-nC1INH at the time of the study start, although additional genes have been identified after the study initiation. Furthermore, as discussed above, mechanisms of vascular leak can be variable in HAE-nC1INH. This variability may also be a factor in idiopathic non-histaminergic angioedema, which may have contributed to the observed study outcome.

## 5 Conclusion

Although the primary efficacy endpoint was not met in CASPIAN, the efficacy of lanadelumab in the studied population of patients with non-histaminergic nC1INH angioedema showed a positive trend in reducing angioedema attack rates. The study outcome is inconclusive given the small sample sizes in some subgroups, high placebo response, and some uncertainty with diagnosis in patients from the idiopathic non-histaminergic angioedema subgroup. The overall long-term data from CASPIAN and CASPIAN OLE suggest a potential clinical benefit with the evidence for symptom control and improved HRQoL. The safety results of the CASPIAN and CASPIAN OLE studies are consistent with the findings from previous studies in patients with HAE Type I/II and confirm that the known safety of lanadelumab is also observed in patients with non-histaminergic nC1INH angioedema.

## Data Availability

The datasets, including the redacted study protocol, redacted statistical analysis plan, and individual participants’ data supporting the results reported in this article, will be made available within 3 months from initial request to researchers who provide a methodologically sound proposal. The data will be provided after its de-identification, in compliance with applicable privacy laws, data protection, and requirements for consent and anonymization. Requests to access the datasets should be directed to https://vivli.org/ourmember/takeda/.

## CASPIAN study group

John Anderson: Clinical Center Research of Alabama, LLC, Birmingham, AL, United States; Francesco Arcoleo: Centro Malattie Rare del Sistema Immunitario e Angioedema UOC Patologia Clinica, AOR Villa Sofia, Cervello, Palermo, Italy; Emel Aygören-Pürsün: Universitätsklinikum Frankfurt Goethe Universität Kinderklinik, Frankfurt, Germany; Aleena Banerji: Massachusetts General Hospital, Boston, MA, United States; Alan P. Baptist: University of Michigan Allergy and Immunology Specialty Clinic, Ann Arbor, MI, United States; Jonathan A. Bernstein: Bernstein Clinical Research Center, LLC, Cincinnati, OH, United States; Danny M. Cohn: Amsterdam University Medical Center, locatie AMC, Amsterdam, the Netherlands; Olivier Fain: Hôpital Saint-Antoine, Paris, France; Henriette Farkas: Semmelweis University, Budapest, Hungary; Pedro Gamboa Setién: Servicio de Alergología, Hospital Universitario Cruces, Bizkaia, Spain; Remi Gagnon: Clinique Spécialisée en Allergie de la Capitale, Québec, QC, Canada; Antonio Gidaro: Azienda Socio Sanitaria Territoriale Fatebenefratelli (Presidio Ospedale Sacco), Milan, Italy; Mar Guilarte: Hospital Universitari vall d’Hebron, Barcelona, Spain; John Hagan: Mayo Clinic, Rochester, MN, United States; Benedikt Hofauer: Klinikum rechts der Isar, Technical University of Munich, Munich, Germany; Joshua S. Jacobs: Allergy & Asthma Clinical Research, Walnut Creek, CA, United States; Marek Jutel: ALL-MED Centrum Medyczne, Medyczny Instytut Badawczy, Wroclaw, Poland; Henry J. Kanarek: Allergy, Asthma & Immunology, Overland Park, KS, United States; Krzysztof Kowal: NZOZ Homeo Medicus, Poradnia Alergologiczna, Białystok, Poland; H. Henry Li: Institute for Asthma & Allergy, Chevy Chase, MD, United States; Michael E. Manning: Asthma & Immunology Associates, LTD, Scottsdale, AZ, United States; Donald McNeil: Optimed Research, LTD, Columbus, OH, United States; James Moy: Rush University Medical Center, Chicago, IL, United States; Yoshiko Oda: Kobe University Hospital, Kobe City, Japan; Hanneke Oude Elberink: University Medical Center Groningen, Groningen, the Netherlands; Daniel Petroni: Seattle Allergy & Asthma Research Institute, Seattle, WA, United States; Marc A. Riedl: University of California San Diego, La Jolla, CA, United States; Heike Röckmann: University Medical Center Utrecht, Utrecht, the Netherlands; Daniel Soteres: Asthma and Allergy PC, Colorado Springs, CO, United States; Giuseppe Spadaro: DAI di Medicina Interna, Immunologia Clinica, Patologia Clinica, Malattie Infettive, Naples, Italy; Petra Staubach: University Medical Center Mainz, Mainz, Germany; Yuzo Suzuki: Clover Hospital, Kanagawa, Japan; Akio Tanaka: Hiroshima University Hospital, Hiroshima, Japan; Massimo Triggiani: UOC Immunologia Clinica e Reumatologia, Azienda Ospedaliera Universitaria San Giovanni di Dio e Ruggi d’Aragona, Salerno, Italy; H. James Wedner: Washington University School of Medicine, St. Louis, MO, United States; William Yang: Ottawa Allergy Research Corporation, Ottawa, ON, Canada; Andrea Zanichelli: Unità Operativa Medicina Generale, Ospedale Luigi Sacco, Milan, Italy.
