# Supplementary material for: Lanadelumab for prevention of attacks of non-histaminergic normal C1 inhibitor angioedema: results from the randomized, double-blind CASPIAN Study and CASPIAN open-label extension
Source: Front Immunol. 2025 May 21;16:1502325. doi: 10.3389/fimmu.2025.1502325 (PMC12135624; doi:10.3389/fimmu.2025.1502325)
Supplement: Supplementary file 1 [file DataSheet1.docx]

**Supplementary Figure S1.** Patient flow in the CASPIAN (**A**) and CASPIAN OLE (**B**) studies.

**Supplementary Figure S2.** Concentration-time profile following lanadelumab 300 mg Q2W in patients with non-histaminergic nC1INH angioedema randomized to lanadelumab in the CASPIAN Study.

**Supplementary Figure S3.** pKal percent change from baseline in patients with non-histaminergic nC1INH angioedema randomized to lanadelumab or placebo in the CASPIAN Study.

**Supplementary Figure S4.** cHMWK percent change from baseline in patients with non-histaminergic nC1INH angioedema randomized to lanadelumab or placebo in the CASPIAN Study.

**Supplementary Table S1.** Inclusion and exclusion criteria for the CASPIAN Study.

**Supplementary Table S2.** Inclusion and exclusion criteria for the CASPIAN OLE Study.
