# Supplementary material for: Lanadelumab for prevention of attacks of non-histaminergic normal C1 inhibitor angioedema: results from the randomized, double-blind CASPIAN Study and CASPIAN open-label extension
Source: Front Immunol. 2025 May 21;16:1502325. doi: 10.3389/fimmu.2025.1502325 (PMC12135624; doi:10.3389/fimmu.2025.1502325)
Supplement: Supplementary file 2 [file DataSheet2.docx]

Supplementary Material

# Supplementary Figures and Tables

## Supplementary Figures


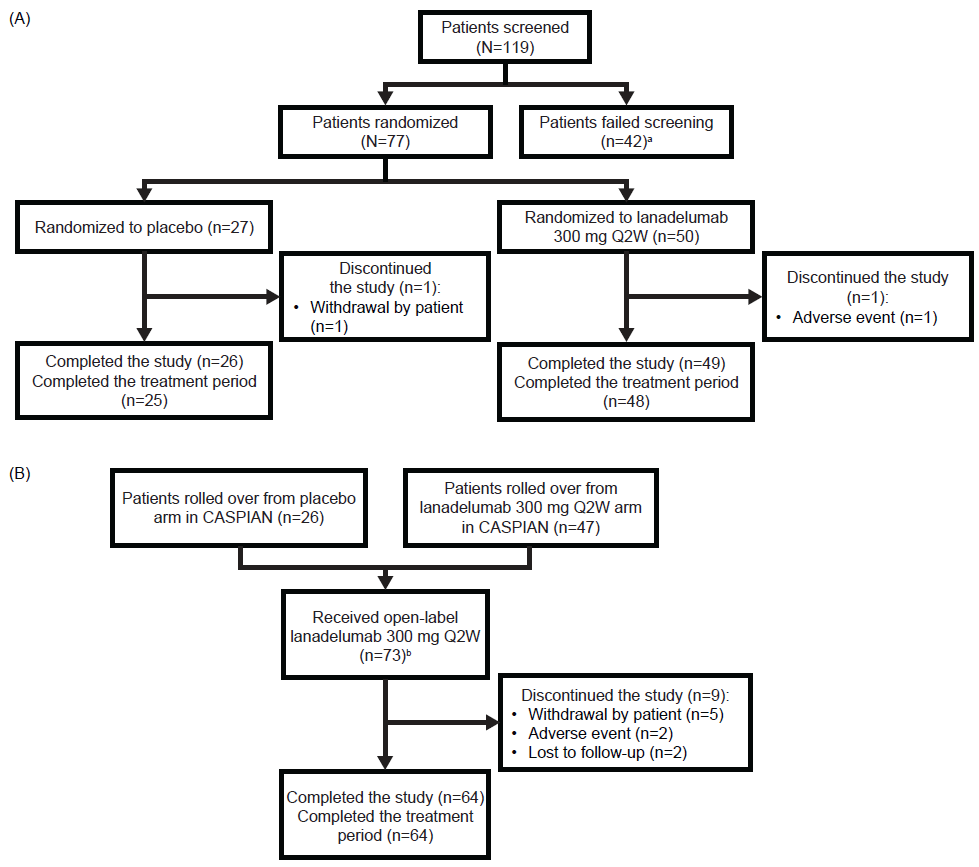


**Supplementary Figure S1.** Patient flow in the CASPIAN (**A**) and CASPIAN OLE (**B**) studies. OLE, open-label extension; Q2W, every 2 weeks. ^a^All patients failed screening by not meeting eligibility criteria. Additional six patients who did not meet eligibility criteria (and therefore were screen failures) participated in and completed the study. ^b^Two patients switched to lanadelumab 300 mg every 4 weeks during the study, and one of these switched back to the Q2W regimen.


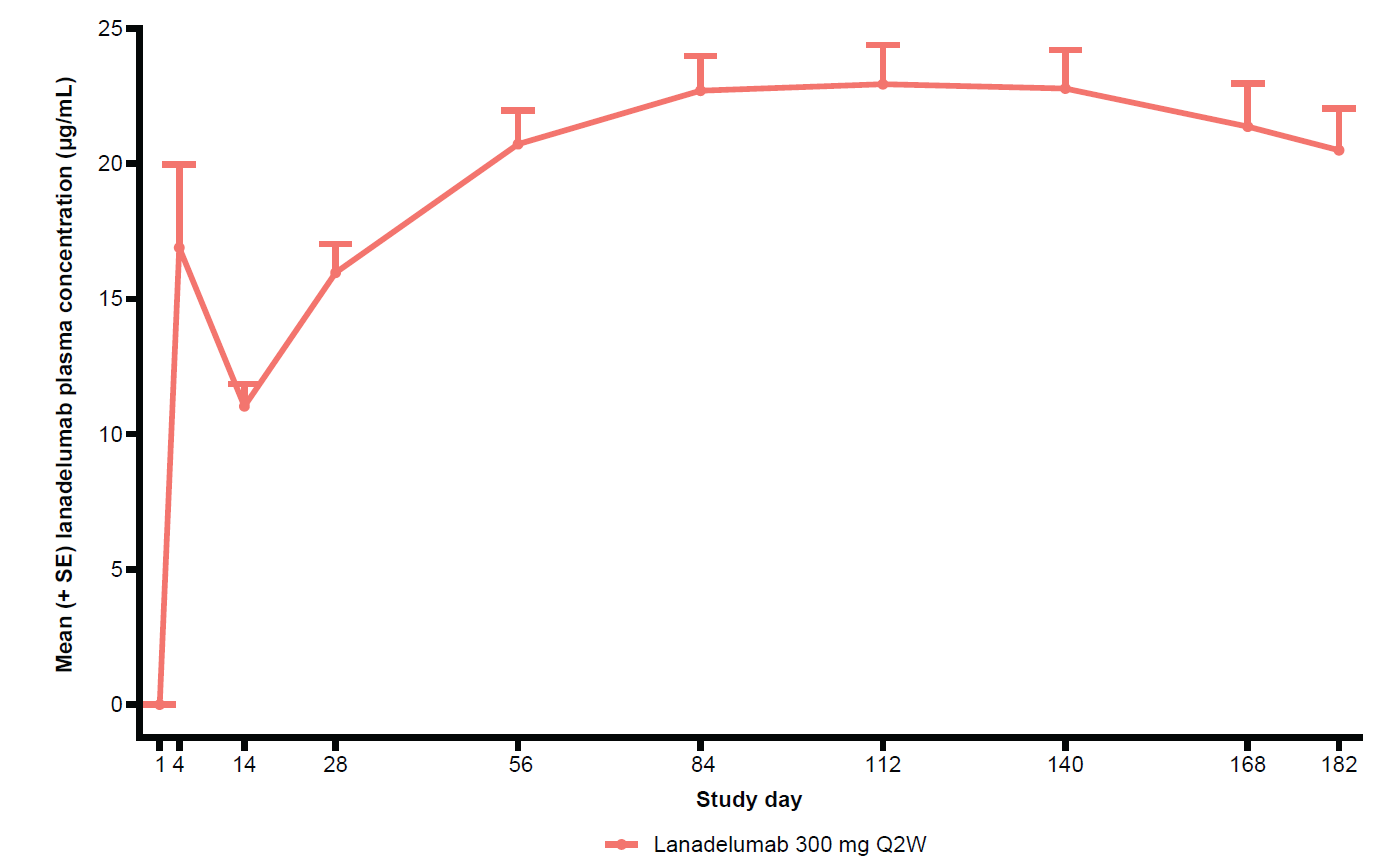


**Supplementary Figure S2.** Concentration-time profile following lanadelumab 300 mg Q2W in patients with non-histaminergic nC1INH angioedema randomized to lanadelumab in the CASPIAN Study. nC1INH, normal C1 inhibitor; Q2W, every 2 weeks; SE, standard error.


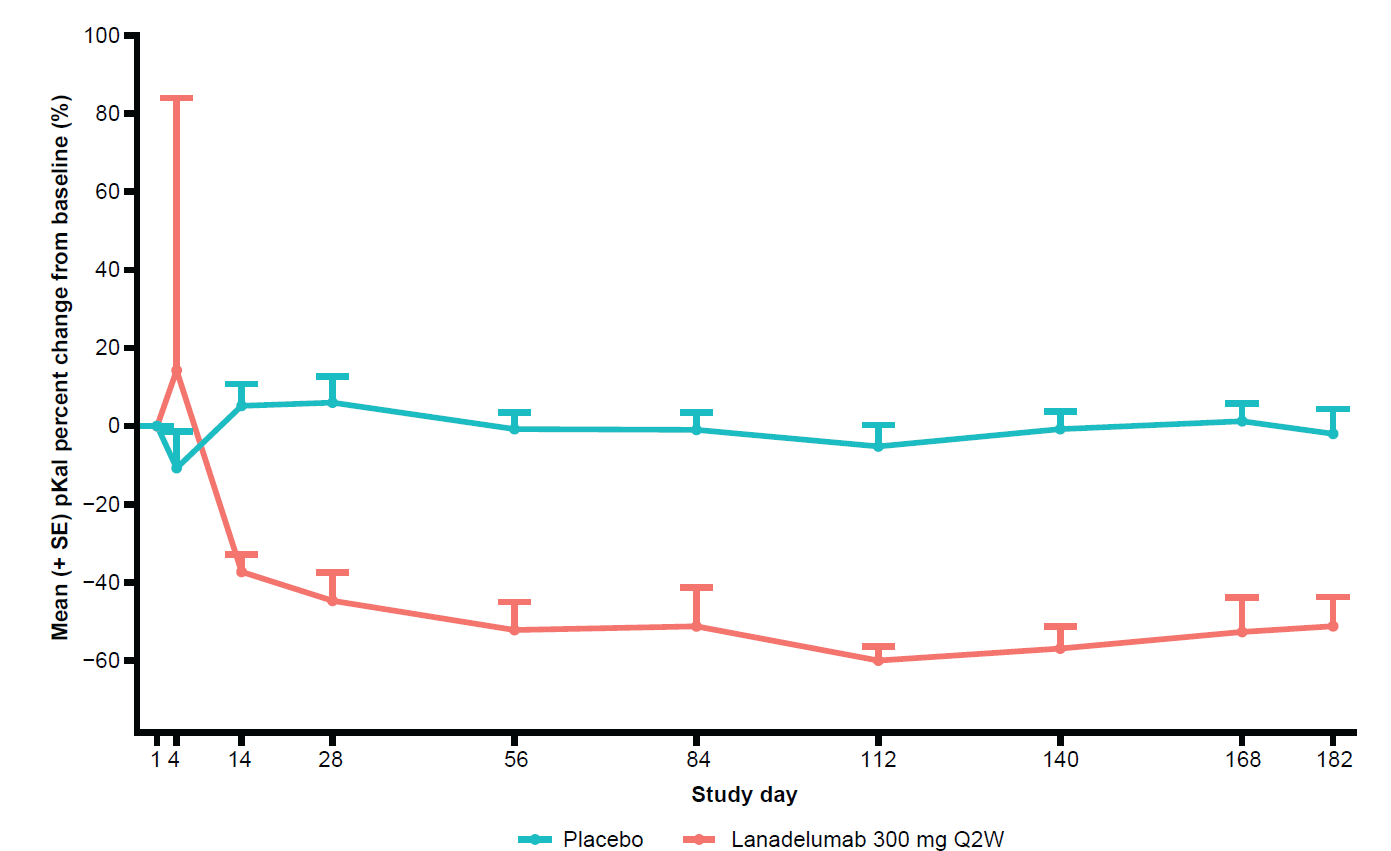


**Supplementary Figure S3.** pKal percent change from baseline in patients with non-histaminergic nC1INH angioedema randomized to lanadelumab or placebo in the CASPIAN Study. nC1INH, normal C1 inhibitor; pKal, plasma kallikrein; Q2W, every 2 weeks; SE, standard error.


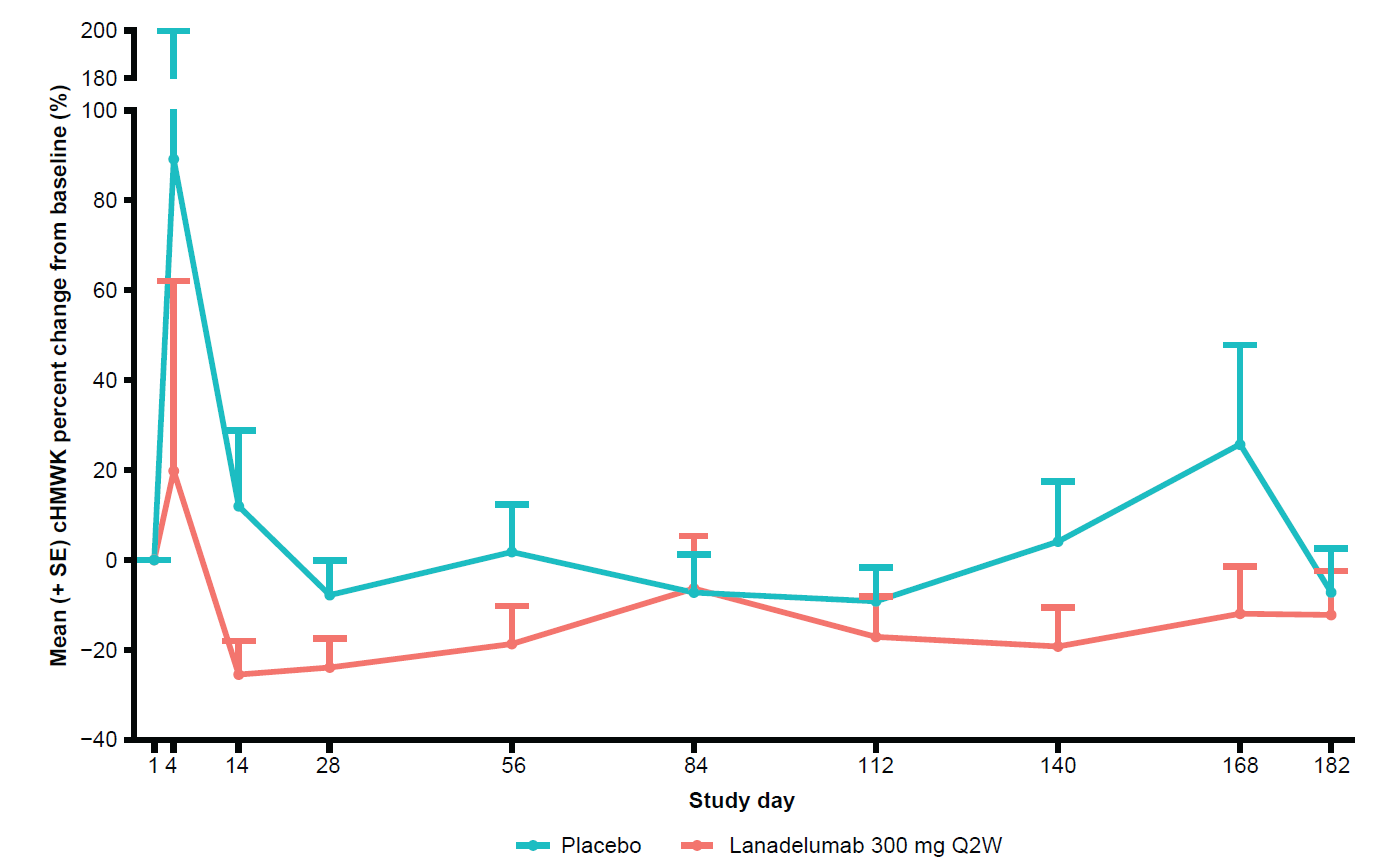


**Supplementary Figure S4.** cHMWK percent change from baseline in patients with non-histaminergic nC1INH angioedema randomized to lanadelumab or placebo in the CASPIAN Study. nC1INH, normal C1 inhibitor; cHMWK, cleaved high-molecular-weight kininogen; Q2W, every 2 weeks; SE, standard error.

## Supplementary Tables

**Supplementary Table S1.** Inclusion and exclusion criteria for the CASPIAN Study.

| **Inclusion criteria** | **Exclusion criteria** |
| --- | --- |
| Males and females, aged ≥12 years for subjects with non-histaminergic nC1INH angioedema at the time of signing of the informed consent form. | Concomitant diagnosis of HAE Type I/II, or recurrent angioedema associated with urticaria. |
| Documented clinical history of recurrent attacks of angioedema in the absence of wheals/urticaria. | Dosing with any investigational drug or exposure to an investigational device within 4 weeks prior to screening. |
| Investigator-confirmed diagnosis of non-histaminergic bradykinin-mediated angioedema with nC1INH as documented by a history of angioedema attack(s) at screening and occurrence of attacks during the observation period:   - History of recurrent angioedema with at least an average of 1 angioedema attack per 4 weeks prior to screening and this attack rate must be confirmed during the observation period while treated with chronic high-dose antihistamine (cetirizine 40 mg/day or equivalent high-dose second-generation antihistamine medication). - Diagnostic testing results obtained during screening from a sponsor-approved central laboratory that confirm C1INH function ≥50% of normal and C4 level not below the normal range. With prior sponsor approval, subjects may be retested during the observation period if results are incongruent with clinical history. - Clinical history of not responding to high-dose antihistamine treatment (cetirizine 40 mg/day or equivalent high-dose second-generation antihistamine medication), which must be confirmed during the observation period with at least 1 angioedema attack per 4 weeks with chronic high-dose antihistamine treatment and no significant difference (as assessed by the investigator and in consultation with the sponsor’s medical monitor, as necessary) from the historic attack rate without high-dose antihistamine treatment. | Exposure to angiotensin-converting enzyme inhibitors or rituximab within 6 months prior to screening. |
| Agree to adhere to the protocol-defined schedule of treatments, assessments, and procedures. | Use of any estrogen-containing medications with systemic absorption (such as oral contraceptives or hormonal replacement therapy) within 4 weeks prior to screening. |
| Subjects aged ≥18 years must be willing to use icatibant as the rescue medication during the observation and treatment period. During the observation period, subjects need to be treated with icatibant for at least 2 angioedema attacks or at least 1 moderate or severe attack. In the opinion of the investigator, subjects with no response to icatibant for acute angioedema attacks in the past medical history/screening, or no improvement or worsened attack severity 2 hours after icatibant treatment during the observation period (based on totality of assessments), will not be included. Note: For subjects aged 12 to <18 years, standard of care therapy per local protocols should be provided. | Response to omalizumab (prophylactic) or corticosteroid (acute/prophylactic) or epinephrine (acute) or leukotriene receptor antagonist (prophylactic) treatments in the past. |
| Males, or non-pregnant, non-lactating females who are of childbearing potential and who agree to be abstinent or agree to comply with the applicable contraceptive requirements of this protocol for the duration of the study. Female subjects of childbearing potential must have a negative serum pregnancy test at screening and must be willing to undergo pregnancy tests throughout the study. Females of non-childbearing potential are defined as surgically sterile (status post hysterectomy, bilateral oophorectomy, or bilateral tubal ligation) or post-menopausal for at least 12 months. | Use of long-term prophylactic therapy for HAE, e.g. C1INH, attenuated androgens (e.g. danazol, methyltestosterone, testosterone), or antifibrinolytics within 2 weeks prior to entering the observation period as long as the investigator determines that doing so would not place the subject at any undue safety risk, and that the subject is at least 18 years of age. |
| The subject (or the subject’s parent/legal guardian, if applicable) has provided written informed consent approved by the institutional review board/research ethics board/ethics committee.  If the subject is an adult, be informed of the nature of the study and provide written informed consent before any study-specific procedures are performed.  OR  If the subject is a minor (i.e. aged <18 years), have a parent/legal guardian who is informed of the nature of the study provide written informed consent (i.e. permission) for the minor to participate in the study before any study-specific procedures are performed. Assent will be obtained from minor subjects. | Any exposure to prophylactic plasma kallikrein inhibitors prior to screening. |
|  | Use of short-term prophylaxis for HAE within 7 days prior to entering the observation period. Short-term prophylaxis is defined as C1INH, attenuated androgens, or antifibrinolytics used to avoid angioedema complications from medically indicated procedures. |
|  | Have any active infectious illness or fever defined as an oral temperature >38°C (100.4°F), tympanic >38.5°C (101.3°F), axillary >38°C (100.4°F), or rectal/core >38.5°C (101.3°F) within 24 hours prior to the first dose of study drug in the treatment period. |
|  | Any of the following liver function test abnormalities: alanine aminotransferase >3× upper limit of normal, or aspartate aminotransferase >3× upper limit of normal, or total bilirubin >2× upper limit of normal (unless the bilirubin elevation is a result of Gilbert’s syndrome). |
|  | Pregnancy or breast feeding. |
|  | Subject has a known hypersensitivity to the investigational product or its components. |
|  | Have any uncontrolled underlying medical condition that would require treatment adjustment during the study treatment period that, in the opinion of the investigator or sponsor, may confound the results of the safety assessments or may place the subject at risk. Subjects with stable treatment for at least 3 months prior to screening and NOT expecting any change to their treatment regimen for 6 months during the study treatment period will not be excluded. |
|  | Have any condition (surgical or medical) that, in the opinion of the investigator or sponsor, may compromise their safety or compliance, preclude the successful conduct of the study, or interfere with interpretation of the results (e.g. significant pre-existing illness or other major comorbidities that the investigator considers may confound the interpretation of study results). |

C1INH, C1 inhibitor; HAE, hereditary angioedema; nC1INH, normal C1 inhibitor.

**Supplementary Table S2.** Inclusion and exclusion criteria for the CASPIAN OLE Study.

| **Inclusion criteria** | **Exclusion criteria** |
| --- | --- |
| Males and females, aged ≥12 years diagnosed with non-histaminergic nC1INH angioedema at the time of enrollment into the antecedent CASPIAN Study. | Discontinued from the CASPIAN Study after enrollment but before Visit 26 for any reason. |
| Subjects must have completed the treatment period (through Visit 26/Day 182) of the CASPIAN Study without reporting a clinically significant TEAE that would preclude subsequent exposure to lanadelumab. | Presence of important safety concerns identified in the CASPIAN Study that would preclude participation in this study. |
| Agree to adhere to the protocol-defined schedule of treatments, assessments, and procedures. | Dosing with an investigational product (not including investigational product defined in the antecedent CASPIAN Study) or exposure to an investigational device within 4 weeks prior to Day 0. |
| Males, or non-pregnant, non-lactating females who are of childbearing potential and who agree to be abstinent or agree to comply with the applicable contraceptive requirements of this protocol for the duration of the study; or females of non-childbearing potential, defined as surgically sterile (status post hysterectomy, bilateral oophorectomy, or bilateral tubal ligation) or post-menopausal for at least 12 months. | Subject has a known hypersensitivity to the investigational product or its components. |
| The subject (or the subject’s parent/legal guardian, if applicable) has provided written informed consent approved by the institutional review board/research ethics board/ethics committee at any time prior to study start. If the subject is a minor (i.e. <18 years of age), have a parent/legal guardian who is informed of the nature of the study provide written informed consent (i.e. permission) for the minor to participate in the study before any study-specific procedures are performed. Assent will be obtained from minor subjects. | Have any condition (surgical or medical) that, in the opinion of the investigator or sponsor, may compromise their safety or compliance, preclude the successful conduct of the study, or interfere with interpretation of the results (e.g. significant pre-existing illness or other major comorbidities that the investigator considers may confound the interpretation of study results). |

nC1INH, normal C1 inhibitor; TEAE, treatment-emergent adverse event.
